# Identification of Frailty Clusters Using Cross-Sectional Frailty and Frailty Trajectory: Cohort of Heart Failure Veterans

**DOI:** 10.1016/j.jacadv.2025.101751

**Published:** 2025-05-28

**Authors:** Javad Razjouyan, Saeed Tofighi, Ariela R. Orkaby, Biykem Bozkurt, Amir Sharafkhaneh, Molly J. Horstman, Parag Goyal, Christopher I. Amos, Orna Intrator, Aanand D. Naik

**Affiliations:** a VA HSR&D, Center for Innovations in Quality, Effectiveness and Safety, Michael E. DeBakey VA Medical Center, Houston, Texas, USA; b Department of Medicine, Baylor College of Medicine, Houston, Texas, USA; c Big Data Scientist Training Enhancement Program, VA Office of Research and Development, Washington, District of Columbia, USA; d Tehran Heart Center, Cardiovascular Diseases Research Institute, Tehran University of Medical Sciences, Tehran, Iran; e New England Geriatrics Research, Education, and Clinical Center, VA Boston Health Care System, Boston, Massachusetts, USA; f Brigham & Women's Hospital, Harvard Medical School, Boston, Massachusetts, USA; g Division of General Internal Medicine, Department of Medicine, Weill Medical College of Cornell University, New York, New York, USA; h Division of Cardiology, Department of Medicine, Weill Medical College of Cornell University, New York, New York, USA; i Department of Internal Medicine, UNM Comprehensive Cancer Center, University of New Mexico, Albuquerque, New Mexico, USA; j Brookdale Department of Geriatrics & Palliative Medicine, Icahn School of Medicine at Mount Sinai, New York, New York, USA; k Geriatrics & Extended Care Data, Analysis Center, James J. Peters VA Medical Center, Bronx, New York, USA; l Department of Management, Policy, and Community Health, The University of Texas Health Science Center at Houston (UTHealth), Houston, Texas, USA; m UTHealth Institute on Aging, University of Texas Health Science Center, Houston, Texas, USA

**Keywords:** cluster analysis, frailty, heart failure, patient outcome, risk assessment

## Abstract

**Background:**

Frailty is a syndrome associated with increased vulnerability and diminished physiological reserves. Three-quarters (78%) of heart failure (HF) patients are frail. Traditional frailty indices (FIs) assess cross-sectional deficits, while frailty trajectories (FTs) measure changes over time.

**Objectives:**

This study aims to examine the interaction between FI and FT to enhance risk stratification in hospitalized adults with HF.

**Methods:**

This retrospective cohort study utilized data from the Veterans Health Administration, including 143,687 veterans aged >50 admitted for HF from 2005 to 2019. FT measurements were derived from FI calculations for each of the 3 years before index hospitalization. Unsupervised clustering identified 4 clusters based on FI and FT interactions: low-low, low-high, high-low, and high-high. Associations between these clusters and clinical outcomes (ie, 1-year mortality, prolonged hospital stays, emergency department visits, and readmissions) were analyzed.

**Results:**

The study cohort was mostly older (mean age 74 ± 10 years), male (98%), and diverse (55% non-Hispanic White). Survival analysis showed distinct mortality risks across clusters; while the 2 clusters with low FI had the longest survival, the high-high group had the lowest survival probability. Adjusted logistic regression indicated that the high-high cluster had over twice the odds of 1-year mortality compared to the low-low cluster (OR: 2.29; 95% CI: 2.15-2.44). The high-high cluster also had significantly higher rates of prolonged hospital stays, emergency department visits, and readmissions at 30 and 90 days postdischarge.

**Conclusions:**

Integrating cross-sectional FI and longitudinal FT offers a comprehensive assessment of frailty in HF patients, improving risk stratification and disease management.

## Central Illustration

**Figure:**
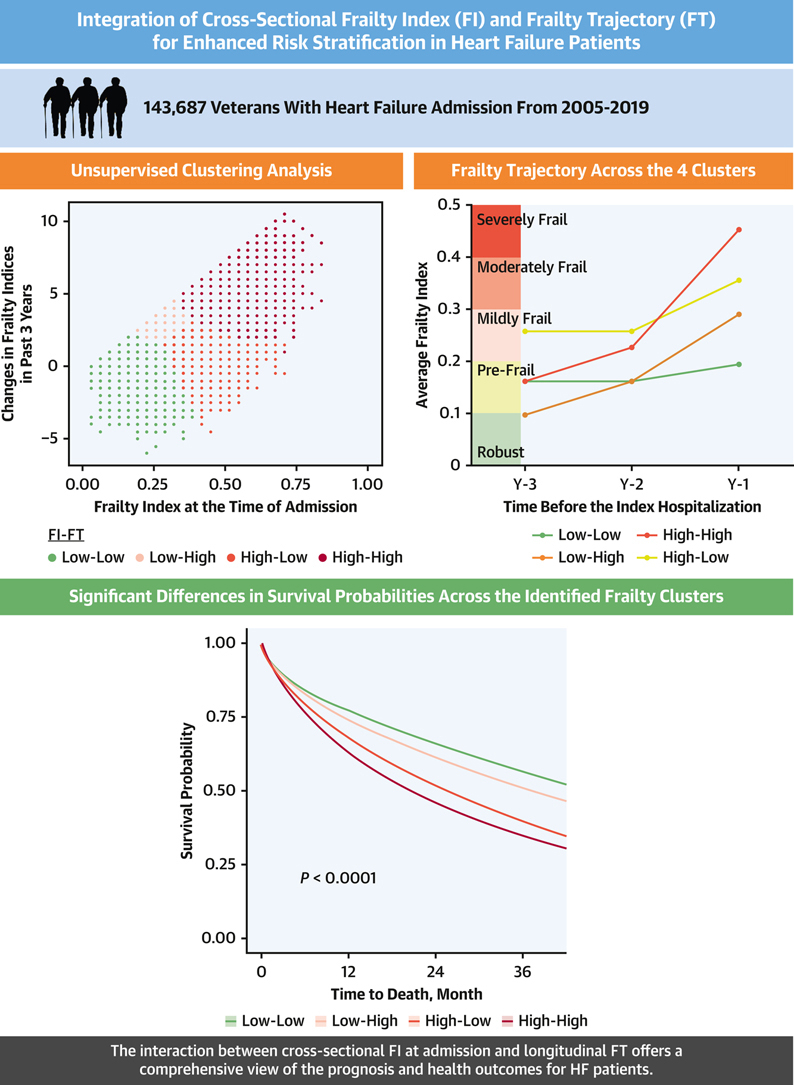

Frailty is a geriatric syndrome of increased vulnerability arising from a loss of physiological reserves and functional abilities across multiple domains of health.^1^ Its role in cardiovascular disease has become increasingly recognized. An estimated 78% of patients with heart failure (HF) are frail.^2^ The accumulation of deficits model is an approach to understanding frailty that measures deficits of physical, psychological, laboratory, and social factors to create a frailty index (FI).^3^ FI scales measure a cross section of the number of variables outside the normal range (deficits) over the total number of variables expressed as a continuous scale from zero (robust) to one (maximum frailty).^4^ Within a FI, deficits can include chronic medical conditions and impairments in sensory and cognitive function.^5^^,^^6^ As deficits accumulate, they compromise an individual's resilience and ability to withstand stressors, leading to increased vulnerability and a heightened risk of adverse health outcomes.^6^, ^7^, ^8^, ^9^ Given the intricate interplay between frailty and adverse outcomes, health systems are integrating automated FI assessment tools within electronic medical record (EMR) systems.^6^^,^^10^^,^^11^

As pragmatic measures of cross-sectional FI became available, new research is reporting on the importance of frailty trajectories (FTs), estimates of FIs across longitudinal time intervals.^12^ Studies on FT find that the progression of frailty over time is associated with a higher risk of adverse outcomes.^12^, ^13^, ^14^, ^15^, ^16^ In contrast with FI, prior studies of FT lack a consistent conceptual framework for guiding FT construction and the integration of FI and FT at the point of care for understanding longitudinal frailty and its association with adverse health outcomes. In the context of frailty, previous studies considered cross-sectional FI and changes in FI as 2 independent variables while in our framework the interaction of these 2 factors is a potential contributor to the future health status of older adults. For instance, 2 older adults admitted to the hospital with HF may have the same level of cross-sectional FI, while one maintained a high cross-sectional FI over the course of years while the other one accumulated several deficits in a short interval. The interactions between a patient’s FT and the cross-sectional FI may affect their immediate and downstream health outcomes.

In this study, we use an unsupervised clustering technique to model the interactions between cross-sectional FI and FT for a population of patients admitted to the hospital for HF. Our aim is to investigate the additive value of combining FI and FT to enhance risk stratification and improve prediction of mortality, hospital readmissions, and emergency department (ED) visits for HF patients.

## Methods

The study’s design and protocol, including all ethical considerations, were approved by Baylor College of Medicine’s Institutional Review Board (H-50294). Additionally, the research was approved by the Michael E. DeBakey Veteran Affairs Medical Center Research and Development Committee.

### Study design and cohort

**Figure 1:**
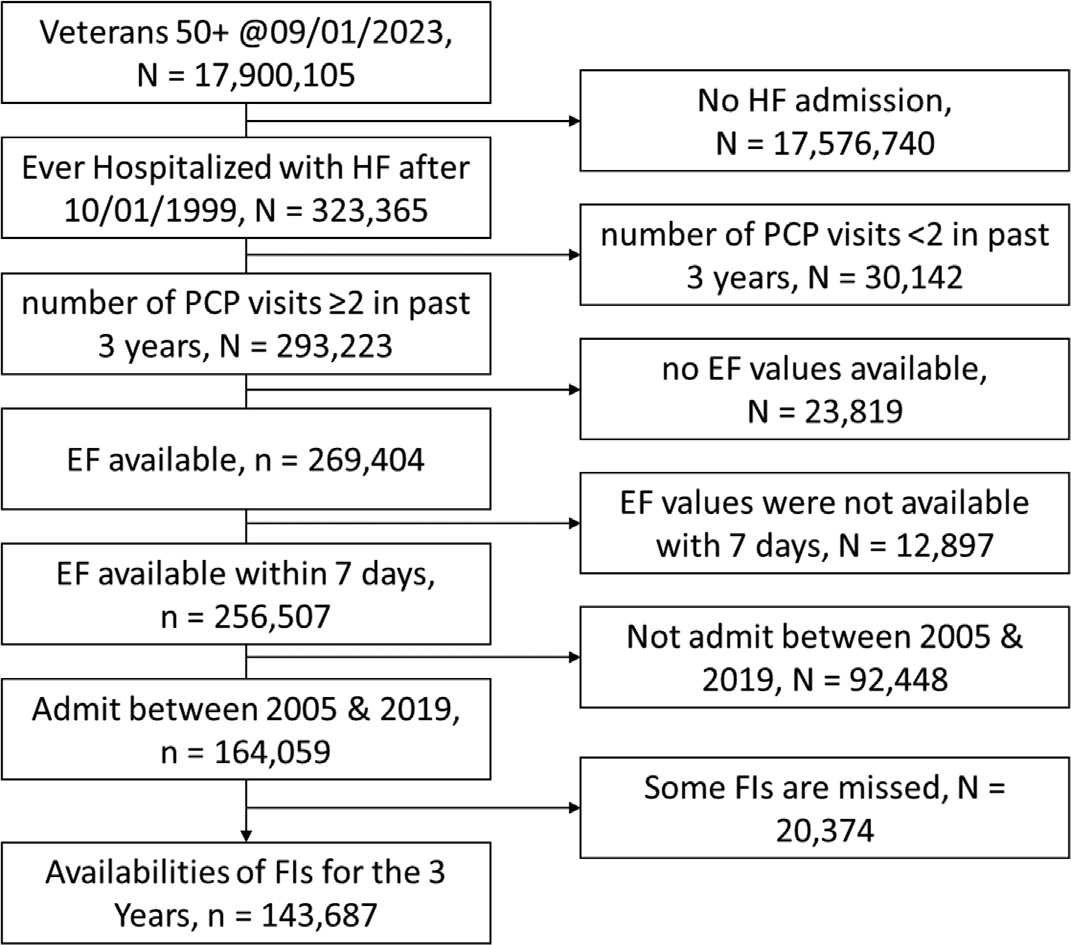
Flowchart Illustrating the Cohort Selection Process From an Initial Population of 17.9 Million Veterans Aged 50 and Above The diagram details the step-by-step inclusion and exclusion criteria based on heart failure admissions, primary care provider (PCP) visits, ejection fraction (EF) data availability, and admission year range, resulting in a final cohort of 146,687 veterans with calculated frailty indices (FIs) over 3 years. HF = heart failure.

This was a retrospective cohort study using national Veterans Health Administration (VA) data. Patient-level data are stored in the VA Corporate Data Warehouse (CDW). CDW is a relational database that contains significant portions of the VA's EMR. We used VA Informatics and Computing Infrastructure which is a remote server that provides software to access the CDW. We included Veterans aged 50 years or older as of January 9, 2023, and limited the cohort to patients admitted with HF as the principal diagnosis after October 1, 1999, when the VA EMR was established. However, we further restricted the analysis to admissions occurring between January 1, 2005, and December 31, 2019. The 2005 starting point was selected because it marked the introduction of the American College of Cardiology and American Heart Association joint guidelines, which were pivotal in shaping modern HF management. The 2019 endpoint ensured data collection was unaffected by the COVID-19 pandemic (Figure 1). Patients with ≥2 primary care physician visits in the past 3 years were included to narrow the sample to primary users of the VA system. We excluded patients without ejection fraction (EF) records during hospitalization and those without any components of VAFI for the prior 3 years before index admission.

### Study measures

#### Outcome variables

The outcome variables included 1-year all-cause mortality, prolonged length of stay (LOS) (≥6 days, which is the third quartile of LOS distribution), ED visits, and all-cause readmissions within 30 and 90 days after discharge from the index of admission.

#### Frailty index

**Figure 2:**
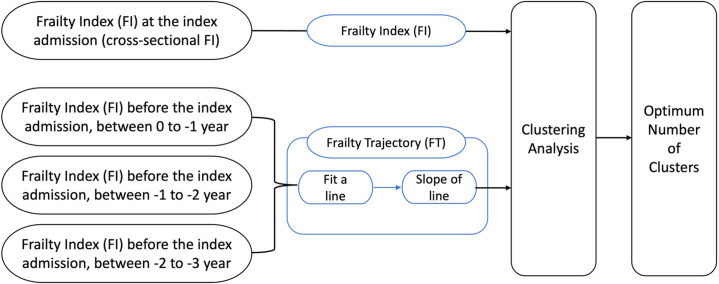
Diagram Depicting the Methodology for Analyzing Frailty Indices (FI) and Frailty Trajectory (FT) in the Study Population Frailty indices are measured at various time points: at the index admission, and up to 3 years prior. These measurements are used to calculate frailty trajectories (FT) by fitting a line and determining its slope. Clustering analysis is then applied to identify the optimum number of clusters for further analysis.

We used the validated VA FI, which captures 31 deficits in health based on International Classification of Diseases-10 and Current Procedural Terminology codes for outpatient visits for the prior 365 days.^6^ To establish a comprehensive view of frailty trends, FI values were calculated for each of the 3 years preceding the index hospital admission for HF. Each calculation covered a 365-day period without overlap between years, ensuring that the data for each FI calculation were independent of prior years. This approach provided a robust framework for identifying frailty trends over time; therefore, International Classification of Diseases or Current Procedural Terminology codes are not automatically transferred from 1 year to another year (<0.1, robust; 0.1-0.2, prefrail; 0.2-0.3, mild frail; 0.3-0.4, moderate frail; ≥0.4, severely frail). The estimated FI at the index date of admission is considered the cross-sectional FI at admission, and we abbreviate it to FI (Figure 2).

#### Frailty trajectory

We measured FT from the integration of the 3 successive FI calculations. We fit a linear line to the 3 calculated FIs for each of the 3 years prior to the index date of admission. While we acknowledge the possibility of nonlinear FTs, we used a linear model for its interpretability and utility for stratifying patients into distinct frailty groups. Additionally, for FT calculation, we intentionally excluded deficits recorded at the time of admission, as they primarily reflect the acute physiological state of the patient during hospitalization and often include transient deficits associated with the acute condition. The rationale for using a 3-year window was based on the consensus of clinician coauthors, who determined that this time frame provided the minimum sufficient amount of data to capture meaningful changes in frailty. We derived a 3-year line from the slope (changes in the FIs) and intercept of this 3-year line. For further analysis, we defined FT as the slope of these 3-year FI changes (Figure 2).

#### Other study variables

We collected age (categorized to <40, 40-65, and ≥65 years), body mass index (BMI) (categorized as obese with BMI ≥30), sex, race (White, Black, and others), and ethnicity (Hispanic) variables from VA Informatics and Computing Infrastructure. We extracted EF using a VA-developed natural language processing tool. The natural language processing tool is validated in VA databases with high positive predictive value (0.97-1.00), sensitivity (0.80-0.90), and specificity (0.92-1.00).^17^ We selected the lowest EF value for patients with multiple records and categorized them as reduced EF (heart failure with reduced EF, ≤40%), mid-range EF (heart failure with modified reduced EF, >40% to <50%), and preserved EF (heart failure with preserved EF, ≥50%).^17^ Charlson comorbidity index (CCI) calculated inpatient and outpatient comorbidities for intervals of 365 days before the index date.^18^ The CCI was calculated annually for 3 consecutive, nonoverlapping years preceding the index admission date.

### Statistical analysis

#### Cluster analysis

**Figure 3:**
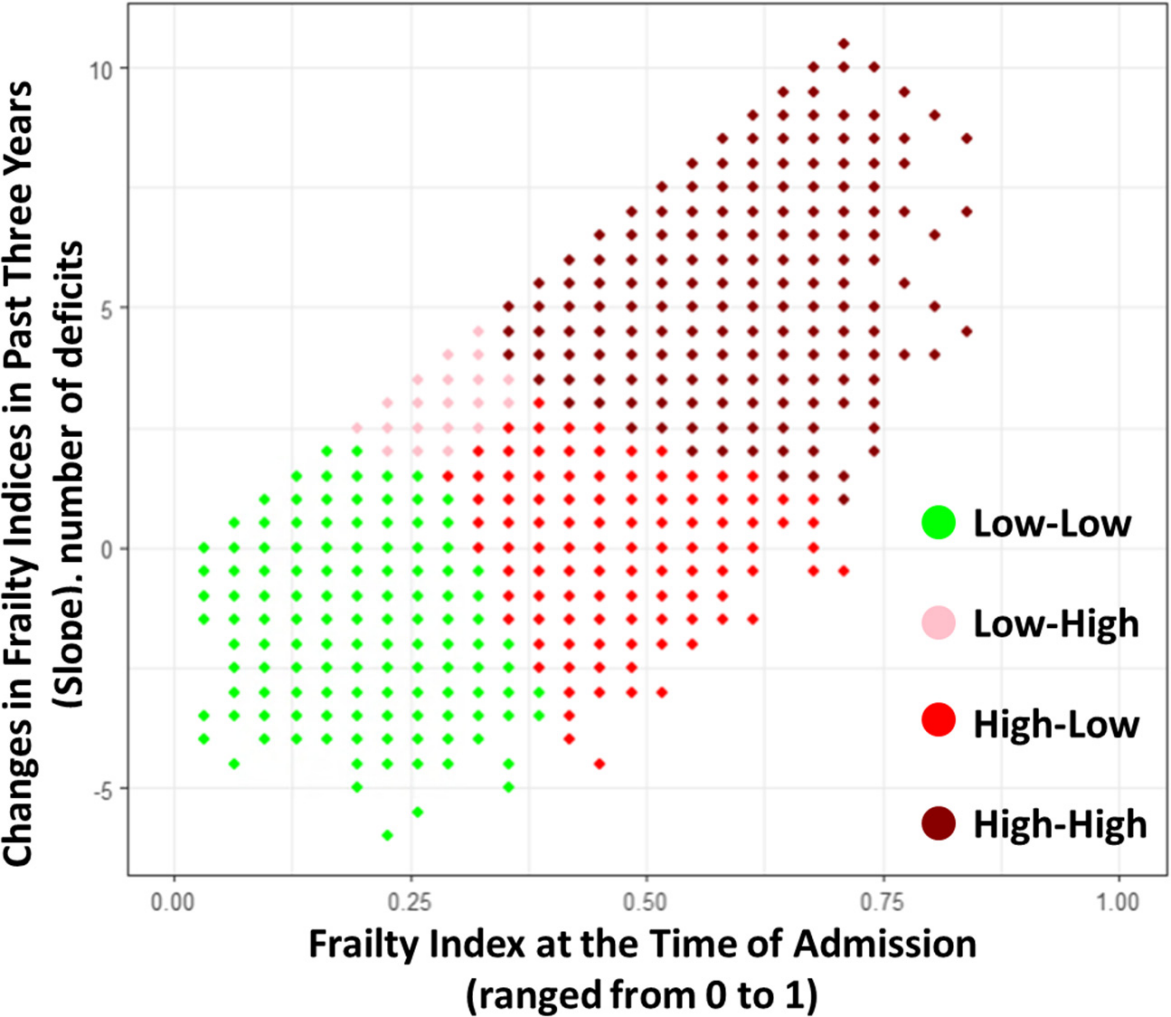
Clustering Analysis of Frailty Trajectories (Changes in FI Over the Past 3 Years) vs the Frailty Index (FI), Revealing 4 Distinct Clusters

We mapped the FI and FT in the two-dimensional variable space, also known as the plane of features. We clustered the interactions between these 2 variables based on the similarity in the plane of features using an unsupervised clustering technique. We used k-means techniques to perform unsupervised clustering due to its simple operation, high compressibility, and scalability for our large data set.^19^^,^^20^ The clusters can represent the emergent properties that are not evident when looking at individual components. Clustering can help identify these properties by showing how collective behaviors arise from the interactions of simpler elements. We employed k-means and identified the optimum number of clusters using the elbow technique. The elbow approach, a commonly used approach in the literature for cluster analysis, provides a visual representation of the optimal number of clusters by plotting the within-cluster sum of squares against the number of clusters.^21^ Although we recognize this technique’s limitations, our decision was guided by choosing an approach that balances statistical rigor with clinical interpretability and ease of implementation in patient management workflows. From this analysis, we identified 4 clusters based on the interactions of FI and the FT (Figure 3), including low-low (low cross-sectional FI and low FT), low-high (low cross-sectional FI and high FT), high-low (high cross-sectional FI and low FT), and high-high (high cross-sectional FI and high FT) (Table 1). All subsequent analyses used these 4 clusters. As mentioned, FT reflects the rate of change in FI over time. In other words, the slope of the FI curve indicates the amount of FT. A steeper slope corresponds to high FT, while a flatter slope corresponds to low FT.

**Table 1:** Demographic Characteristics of the Study Cohort

	All (N = 143,687)	Low-Low (n = 53,593)	Low-High (n = 34,752)	High-Low (n = 30,798)	High-High (n = 24,544)
Age, y	73.5 ± 10.6	71.6 ± 10.7	72.9 ± 10.6	75.5 ± 10.1	75.9 ± 10.3
Age <65 y	32,338 (22.5)	15,538 (29.0)	8,519 (24.5)	4,719 (15.3)	3,562 (14.5)
Age 65-75 y	46,170 (32.1)	17,120 (31.9)	11,167 (32.1)	9,955 (32.3)	7,928 (32.3)
Age 76-85 y	38,874 (27.1)	13,378 (25.0)	9,209 (26.5)	9,218 (29.9)	7,069 (28.8)
Age ≥85 y	26,305 (18.3)	7,557 (14.1)	5,857 (16.9)	6,906 (22.4)	5,985 (24.4)
Male	140,993 (98.1)	52,659 (98.3)	34,172 (98.3)	30,147 (97.9)	24,015 (97.8)
Race					
White	99,499 (69.2)	35,400 (66.1)	23,657 (68.1)	22,437 (72.9)	18,005 (73.4)
Black	27,348 (19.0)	11,578 (21.6)	6,840 (19.7)	4,947 (16.1)	3,983 (16.2)
Others	9,214 (6.4)	3,467 (6.5)	2,337 (6.7)	1,897 (6.2)	1,513 (6.2)
Ethnicity, Hispanic	6,676 (4.6)	2,572 (4.8)	1,588 (4.6)	1,489 (4.8)	1,027 (4.2)
BMI, kg/m^2^	31.0 ± 7.8	31.2 ± 7.9	30.7 ± 7.7	31.3 ± 7.8	30.3 ± 7.6
BMI ≥30 kg/m^2^	69,540 (48.4)	26,679 (49.8)	16,285 (46.9)	15,598 (50.6)	10,978 (44.7)
EF	40 (25-55)	40 (25-50)	40 (25-55)	40 (30-55)	40 (30-55)
HFrEFn	82,500 (57.4)	32,891 (61.4)	20,297 (58.4)	16,022 (52.0)	13,290 (54.1)
HFmEFn	11,474 (8.0)	3,915 (7.3)	2,775 (8.0)	2,749 (8.9)	2,035 (8.3)
HFpEFn	49,713 (34.6)	16,787 (31.3)	11,680 (33.6)	12,027 (39.1)	9,219 (37.6)
CCI	4.4 ± 2.6	3.3 ± 2.1	3.8 ± 2.3	5.6 ± 2.6	5.8 ± 2.9
CCI 2+	127,074 (88.4)	43,470 (81.1)	30,149 (86.8)	29,909 (97.1)	23,546 (95.9)

Values are mean ± SD, n (%), or median (IQR).

BMI = body mass index; CCI = Charlson comorbidity index; EF = ejection fraction; HFmEF = heart failure with modified reduced EF; HFpEF = heart failure with preserved EF; HFrEF = heart failure with reduced EF; high-high = high cross-sectional FI and high frailty slope; high-low = high cross-sectional FI and low frailty slope; low-high = low cross-sectional FI and high frailty slope; low-low = low cross-sectional FI and low frailty slope.

After establishing clusters based on the interactions between FI and FT, we compared sociodemographic and clinical variables by cluster. For descriptive characteristics of the cohort, we presented continuous variables as mean ± SD and categorical variables as number (percentage). For categorical variables, we used Fisher exact test to compare between clusters. For continuous variables, we used unpaired *t*-test to compare between groups. The significance level was *P* value <0.05.

We report Kaplan-Meier curves and the Cox proportional HRs to model differences in the probability of death among the clusters. We consider the cluster low-low as the reference. To report the general performance of FT vs FI, we conducted 3 regression analyses considering all-cause mortality as the dependent variable and FI, FT, and FI + FT as the independent predictors. We reported the area under the curve for each model. We conducted unadjusted and adjusted analyses to determine the association of clusters with adverse outcomes, including 1-year all-cause mortality, prolonged hospital stay, ED visits, and all-cause hospital readmission. We report the ORs and 95% CIs using logistic regression and adjusted with age, sex, race, ethnicity, BMI, and EF. Logistic regression was employed for the majority of outcomes due to its suitability for analyzing binary variables. However, for 3-year mortality, we opted for a survival analysis approach as it accounts for time-to-event data, providing a more nuanced understanding of mortality risks over an extended period.

## Results

### Characteristics of study population

We assembled a cohort of 143,687 veterans with index HF admissions (see Figure 1). Table 1 describes the characteristics of the study population. The mean age was 74 ± 10 years, 98% were male, (69% White and 19% Black), obese (BMI 30 ± 8 Kg/m^2^), and 52% had an EF >40%. Patients have significant multimorbidity (CCI at index date was 4.9 ± 2.9; 91% had CCI ≥2). Mortality rate over the subsequent 3 years was high, 70.1%, with a median time to death of 1.5 (IQR: 0.5-3.1) years postdischarge.

### Clusters: general

**Figure 4:**
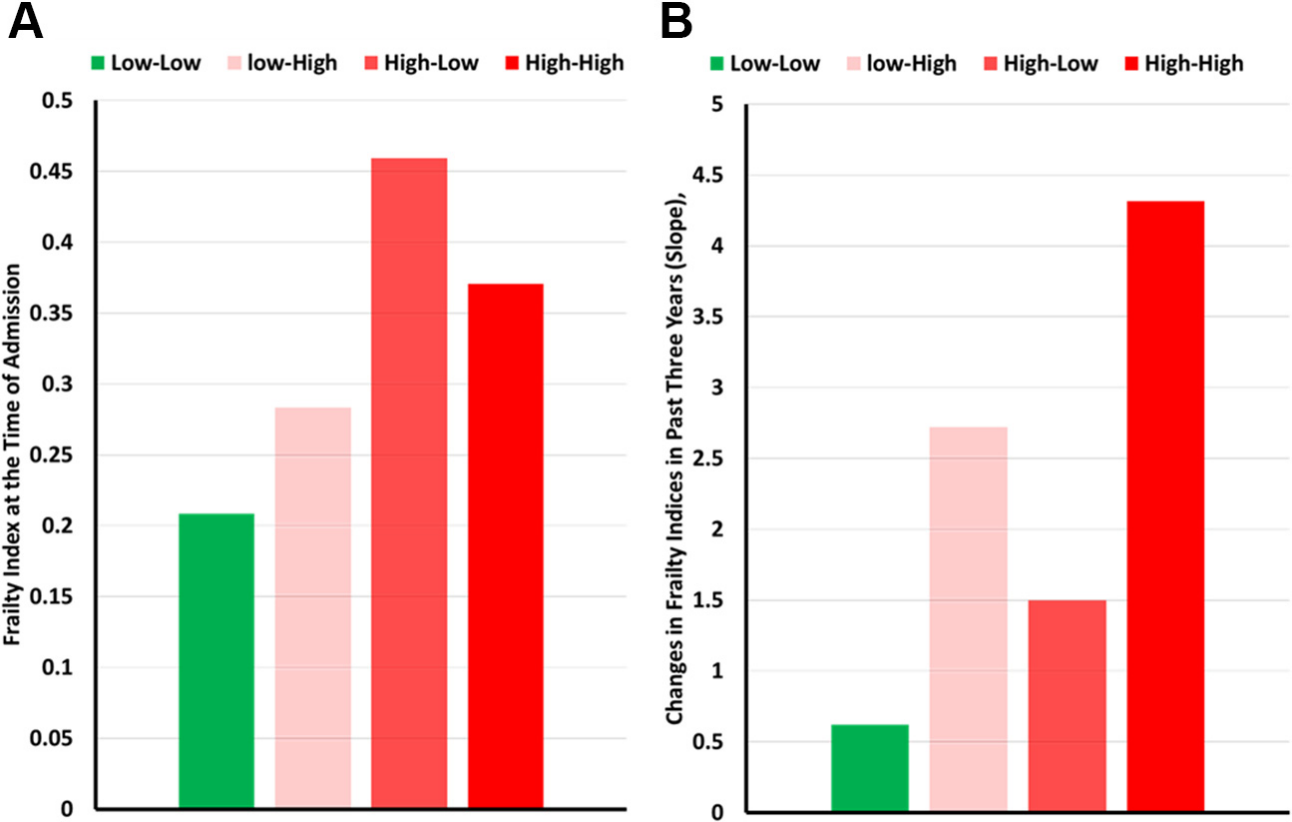
Comparing Frailty Index and Frailty Trajectory Changes Between 4 Study Clusters Bar graphs comparing the frailty index at the main hospitalization (A) and the frailty trajectory (B) across the 4 study clusters.

**Figure 5:**
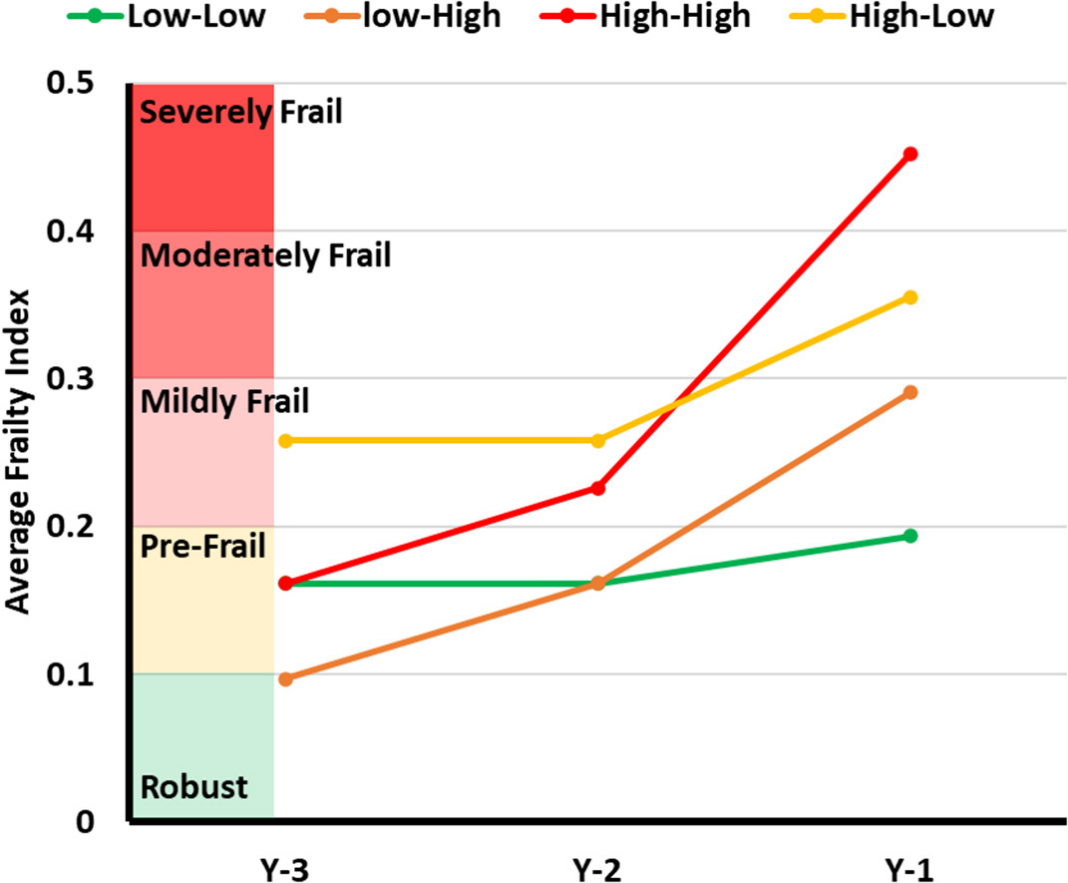
Comparison of Frailty Trajectory Over the 3 Years Leading up to the Index Hospitalization Across 4 Study Clusters, Illustrating the Changes in Average Frailty Indices Within Each Cluster

The algorithm categorized the sample into 4 clusters based on FI and FT values. The median cutoff score for FI is 0.3, equivalent to moderate frailty. The median cutoff score for FT is 0.03, equivalent to one new deficit per year. These 4 clusters, illustrated in Figures 4 and 5, include low-low (FI = 0.198, FT = 0.019); low-high (FI = 0.29, FT = 0.078); high-low (FI = 0.367, FT = 0.015); and high-high (FI = 0.455, FT = 0.126). The high-low group had the lowest prevalence (n = 7,348, 15%) while the high-high group had the highest (n = 18,487, 39%). The low-low group was younger (71.0 ± 10.6) compared to low-high (73.1 ± 10.4), high-low (75.3 ± 10.2), and high-high (75.8 ± 10.2), Table 1. The high-high had the lowest proportion of BMI ≥30 kg/m^2^ (38.0%) compared to low-low (46.0%), low-high (42.7%), and high-low (44.2%).

### Clusters—mortality

**Figure 6:**
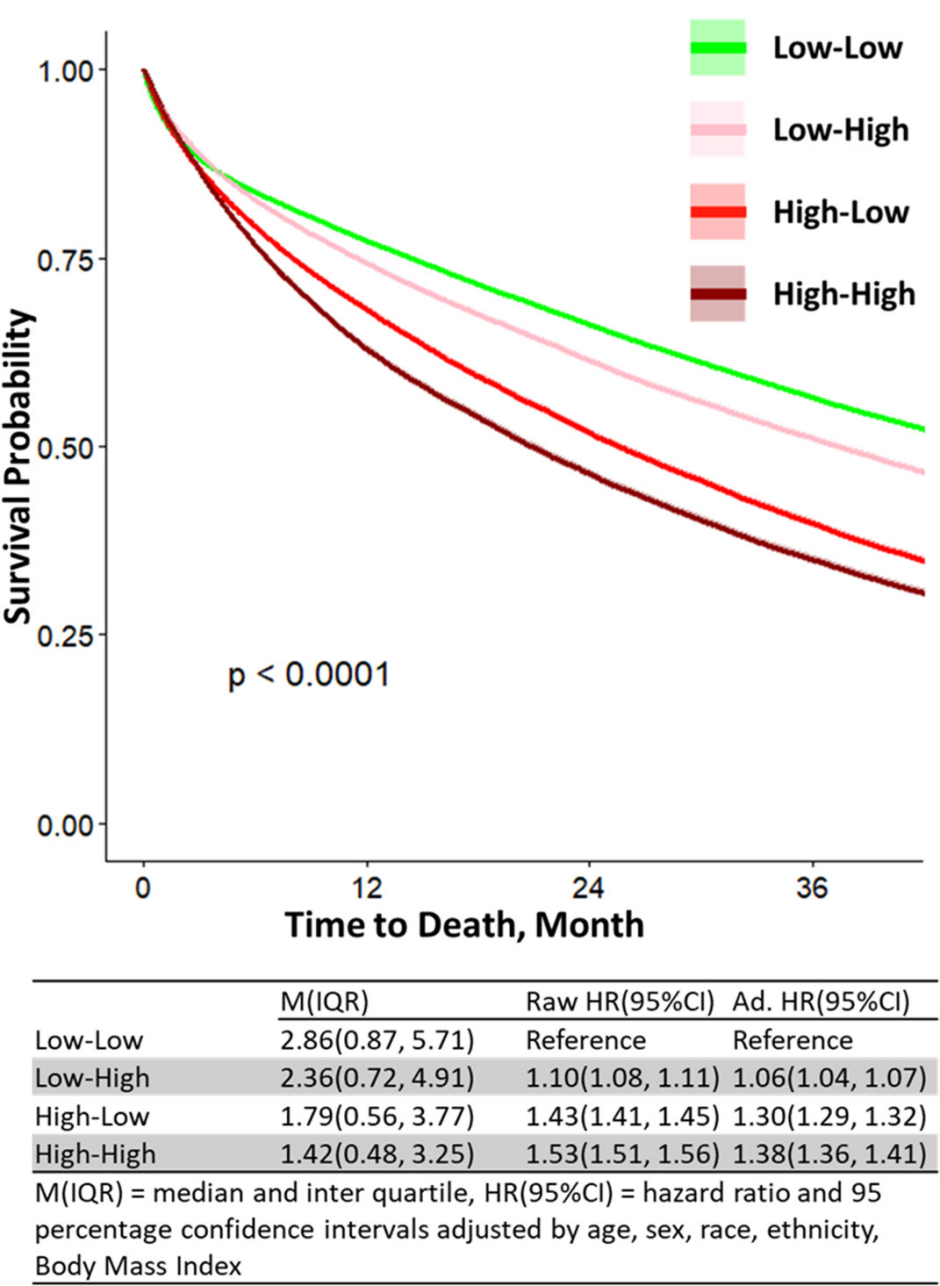
Kaplan-Meier Analysis Shows the Survival Probability Over 36 Months Following the Index Hospitalization Across 4 Study Clusters A progressively significant increase in mortality is observed across the clusters compared to the low-low group.

The survival analysis demonstrates 4 distinct clusters with nonoverlapping survival curves over 36 months (Figure 6). The 2 clusters with low FI (low-low and low-high) had the longest median survival (25 and 22 months, respectively). When compared with low-low each of the other 3 clusters had significantly higher mortality rates. There is a step-wise significant increase in mortality across clusters when compared with low-low: (low-high OR: 1.09 [95% CI: 1.05-1.13]; high-low OR: 1.29 [95% CI: 1.24-1.34]; high-high OR: 1.35 [95% CI: 1.31-1.40]). The adjusted, multivariate logistic regression analysis demonstrates similarly significant increases in 1-year mortality across the 3 categories when compared with low-low (see Table 2, top rows). The high-high category had a >2-fold increased odds of death (OR: 2.29 [95% CI: 2.15-2.44]) at 1 year compared to low-low.

**Table 2:** Relationship of Frailty Clusters and Clinical Outcomes

	N (%)	Raw OR (95% CI)	Adj. OR (95% CI)
1-y all-cause mortality			
Low-low	45,213 (84.4)	Reference	Reference
Low-high	29,642 (85.3)	1.08 (1.04-1.12)	1.01 (0.97-1.05)
High-low	27,723 (90.0)	1.67 (1.60-1.75)	1.40 (1.34-1.46)
High-high	22,025 (89.7)	1.62 (1.55-1.70)	1.32 (1.26-1.39)
Prolonged length of stay			
Low-low	12,348 (23.0)	Reference	Reference
Low-high	10,652 (30.7)	1.48 (1.43-1.52)	1.48 (1.44-1.53)
High-low	9,210 (29.9)	1.43 (1.38-1.47)	1.44 (1.39-1.48)
High-high	9,146 (37.3)	1.98 (1.92-2.05)	1.99 (1.93-2.06)
ED visit in 30 d postdischarge			
Low-low	6,771 (12.6)	Reference	Reference
Low-high	6,568 (18.9)	1.61 (1.55-1.67)	1.07 (1.06-1.07)
High-low	6,950 (22.6)	2.02 (1.94-2.09)	1.11 (1.10-1.11)
High-high	7,114 (29.0)	2.82 (2.72-2.93)	1.18 (1.17-1.19)
ED visit in 90 d postdischarge			
Low-low	13,647 (25.5)	Reference	Reference
Low-high	12,403 (35.7)	1.62 (1.58-1.67)	1.11 (1.10-1.12)
High-low	13,445 (43.7)	2.27 (2.20-2.34)	1.20 (1.20-1.21)
High-high	13,365 (54.5)	3.50 (3.39-3.61)	1.34 (1.33-1.35)
Hospital readmission in 30 d postdischarge			
Low-low	8,480 (15.8)	Reference	Reference
Low-high	8,536 (24.6)	1.73 (1.67-1.79)	1.09 (1.09-1.10)
High-low	8,423 (27.3)	2.00 (1.94-2.07)	1.12 (1.12-1.13)
High-high	8,938 (36.4)	3.05 (2.94-3.15)	1.23 (1.22-1.24)
Hospital readmission in 90 d postdischarge			
Low-low	14,429 (26.9)	Reference	Reference
Low-high	14,003 (40.3)	1.83 (1.78-1.89)	1.15 (1.14-1.15)
High-low	13,969 (45.4)	2.25 (2.19-2.32)	1.21 (1.20-1.22)
High-high	14,409 (58.7)	3.86 (3.74-3.98)	1.39 (1.38-1.40)

ED = emergency department; other abbreviations as in Table 1.

The analysis indicates a progressive increase in mortality odds across the groups from low-low to low-high to high-low to high-high. These findings suggest that while FT offers additional predictive value, the FI appears to play a more significant role in predicting mortality.

### Clusters: health outcomes

For associations with prolonged hospital stays (6 or more days), there was a nonsignificant difference between low-high and high-low clusters. Both clusters, however, have significantly greater associations with prolonged stays compared with low-low and significantly fewer compared with high-high. The magnitude of differences in associations with ED or hospital readmissions is lower across clusters but they are significantly different. In particular, ED visits and hospital readmissions have about 20% higher odds at 30 days and 30% higher odds at 90 days for the high-high compared with low-low clusters (Table 2, bottom rows).

## Discussion

In this large national cohort study of more than 143,000 older adults hospitalized with HF, we identified frailty clusters based on the interactions between FI and FT using an unsupervised clustering algorithm. The clusters differed based on FI at the time of admission and FT over the past 3 years, respectively, creating 4 distinct categories: (low-low, low-high, high-low, and high-high). In survival analysis, these clusters provide distinct trajectories for predicting mortality at 1 year and over a 3-year course. We observed that FI at the time of admission is the most predictive of mortality while FI in combination with the FT predicts the health care utilizations. The clusters also differed significantly in their associations with the health outcomes of prolonged hospital LOS, and ED visits and readmissions at 30 days and 90 days. The 2 mixed clusters (low-high and high-low) were similar in their associations with 30-day ED visits and readmissions, but both were significantly different from low-low and high-high clusters. These findings suggest that the integration of FI and FT data provides clinically meaningful data on mortality and adverse health outcomes beyond traditional cross-sectional FIs.

In this study, we considered the interaction of FI and FT as the best approach to identify the clusters of patients. In a Chinese Longitudinal Healthy Longevity and Happy Family Study of 4,083 participants, authors used unsupervised clustering to identify 2 distinct trajectories: stable-growth and rapid-growth. The authors used other machine learning algorithms to identify factors dedicated to the progression of frailty but did not report health care outcomes associated with the 2 clusters.^22^ In a study among 3 population-based cohorts (N = 3,689), the authors reported that FI was a more significant factor in risk stratification than FT.^16^ Our study showed that capturing both FI and FT will improve the prediction of adverse health outcomes. Another study on older adults 75+ (N = 508) showed that the rate of FI change was more important than cross-sectional FI for short-term mortality prediction.^23^ In contrast, our study showed that the interaction between FI and FT and the associated clusters predicted different odds of mortality at 1 year and over a 3-year survival analysis. Another study from 4 different cohorts of community-dwelling older adults aged 65+ confirmed prior studies showing FT predicted mortality independently of cross-sectional FI.^24^ The authors highlighted that repeated assessment of frailty and an individual’s frailty trajectory could provide a means to anticipate further deterioration and mortality among older adults. Our study shows that FI changes (FT) will add an additional dimension to the cross-sectional FI in the form of distinct clusters of older patients. Additionally, in another recently published analysis by our team on a subgroup of veterans with HF (N = 54,774), we observed that incorporating longitudinal FT significantly enhanced 1-year mortality prediction by up to 24% among patients in the prefrail range, compared to using FI alone. In the overall population, however, we observed a more modest improvement of at least 4.1% in 1-year mortality prediction.^25^

Frailty assessment is important in HF patients because it provides valuable prognostic information and helps guide clinical decision-making.^26^, ^27^, ^28^ Frailty is associated with poor clinical outcomes in HF patients, including increased mortality and longer hospital stays.^29^ Future studies should consider the role of frailty assessment (using FI and FT) to identify patients who may benefit from specific therapies, such as mechanical circulatory support and transplantation, as frailty in HF patients is potentially modifiable. Additionally, frailty assessment plays a crucial role in risk stratification, treatment planning, and improving outcomes in HF patients.^30^^,^^31^ The additive value of FT to the existing frailty literature in HF patients is the identification of clusters that may react to treatment differently (low-low vs low-high) or experience higher rates of prolonged hospital stays (high-low vs high-high) or more frequent health services utilization (readmission rates). Further research exploring the role of frailty clusters in predicting better responses to high intensity but critical treatments, such as mechanical circulatory support and transplantation are needed.^32^

Our study has limitations. We did not include the patients’ data from the Centers for Medicare and Medicaid Services although we ensured the continuum of care in the VA health care system by enforcing ≥2 primary care provider visits in the past 36 months before the index date of admission. The sample is predominately male but does include a diverse population by race, ethnicity, and geographic distribution. Study results did not consider medication or type of treatment during hospitalization as a possible confounding factor for mortality or health care utilization trends.

## Conclusions

In summary, methods for calculating frailty provide useful predictions of adverse outcomes among adults with HF. The addition of FT data improves predictions among older Veterans with HF. The interaction between FI at the time of admission and FT provides a comprehensive picture regarding the prognosis and health outcomes of patients with HF (Central Illustration).

Perspectives **COMPETENCY IN MEDICAL KNOWLEDGE:** Our study reveals 4 distinct frailty clusters in older adults hospitalized with HF by combining FI and FT analyses. The findings highlight that integrating FI and FT significantly improves risk stratification and predicts mortality and adverse outcomes more effectively than using FI alone. Clinicians can utilize this approach to better identify high-risk patients and tailor treatment plans, potentially leading to more effective and targeted interventions.

**TRANSLATIONAL OUTLOOK:** Future research should investigate how different frailty clusters respond to various HF treatments to develop cluster-specific protocols. Barriers to implementation may include the need for standardized frailty assessment methods and integrating these tools into routine practice.

**Central Illustration:**
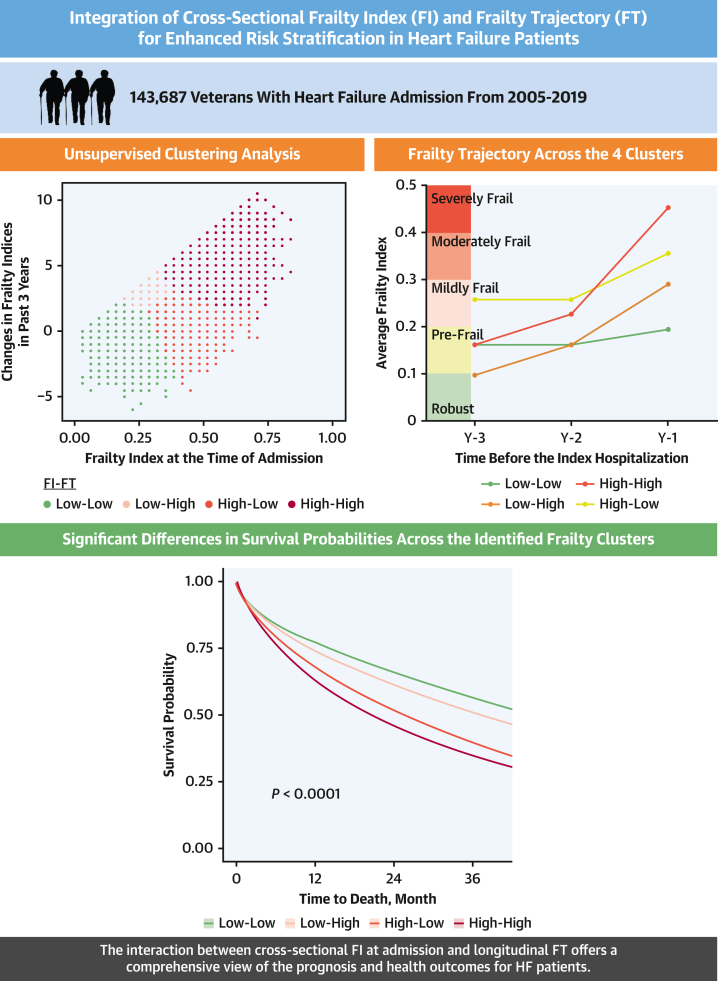
Integrating the Cross-Sectional FI at Admission With Longitudinal FT Enhances Risk Stratification in Heart Failure Patients Among the 4 frailty clusters, the high-high cluster begins at a prefrail state but, due to rapid deterioration (high FT), progresses to severely frail, resulting in the highest FI observed. These distinct patterns correspond to significant differences in survival outcomes, emphasizing the importance of both FI and FT in predicting patient prognosis.

## Funding support and author disclosures

The analysis was supported by seed funding from 10.13039/100007856Baylor College of Medicine at Houston, Texas; the Center for Innovations in Quality, Effectiveness, and Safety (CIN 13-413); Michael E. DeBakey VA Medical Center, Houston, Texas; and a 10.13039/100000002National Institutes of Health (NIH), 10.13039/100000050National Heart, Lung, and Blood Institute (NHLBI) K25 funding (#:1K25HL152006-01). Dr Horstman is supported by a Department of Veterans Affairs Health Services Research and Development Career Development Award (CDA 21-143). All other authors have reported that they have no relationships relevant to the contents of this paper to disclose.
